# Tetanus Toxoid Immunization Status and Associated Factors among Mothers in Damboya Woreda, Kembata Tembaro Zone, SNNP, Ethiopia

**DOI:** 10.1155/2018/2839579

**Published:** 2018-11-22

**Authors:** Muluken Dubale Mamoro, Lolemo Kelbiso Hanfore

**Affiliations:** ^1^Damboya Woreda, Curative and Rehabilitative Core Process Coordinator of the Health Office, SNNP, Ethiopia; ^2^Wolaita Sodo University, College of Health Sciences and Medicine, Department of Nursing, Wolaita Sodo, Ethiopia

## Abstract

**Background:**

Tetanus toxoid immunization is one of the proven strategies for eliminating maternal and neonatal tetanus. According to Ethiopian Demographic Health Survey of 2016, only 49% of mothers received two tetanus toxoid (TT) injections during their last pregnancy which is below the World Health Organization and Ethiopia Ministry of Health recommendation. Therefore, the aim of this study was to determine the status of TT immunization among mothers in Damboya Woreda, South Ethiopia.

**Method:**

A community-based cross-sectional study was conducted from March 1 to 26, 2017, in Damboya Woreda. A total of 837 mothers who had given birth in the last 12 months were included in the study. The simple random sampling method was used to select the study participants, and data were collected through an interview using a structured questionnaire. Data were entered into Epi data software version 3.1 and exported to SPSS version 22 for further analysis. Logistic regression was used to identify independent predictors of the immunization status of mothers at a 5% significance level.

**Result:**

The finding of this study revealed that 607 (72.5%) mothers were protected at birth against tetanus. Age of mother who attended elementary school, husbands' education status, visited by HEW's at home, making joint health decision with husband, use of modern family planning method, number of antenatal care visit, and time to reach the nearest health facility were independent predictors of TT immunization status among the mothers.

**Conclusion:**

Significant proportions of the mothers were not taken at least two doses of TT vaccine which is a minimum dose to prevent maternal or neonatal tetanus. Even though most mothers had access for TT immunization service, they were not immunized with protective doses of TT vaccine.

## 1. Introduction

Tetanus vaccine is an inactivated toxin (toxoid) that was produced in 1924 and became commercially available in 1938 [1]. The immunization of pregnant women or women of childbearing age with two doses of TT vaccination may reduce the neonatal tetanus mortality by 94% [2].

Globally, an estimated 3.3 million neonatal deaths occur each year and about 9,000 babies who were within the first 28 days of life die each day. From this death, NT shares a high number [3], and in 2015, WHO estimate 34,019 newborns died from NT [4].

A study done in Ghana revealed that 71% of mothers had received two doses of TT injection during pregnancy or protected at birth [5], and another study in Kenya showed 63% mothers had received TT2+ doses of injection during their last child pregnancy [6].

Concerning maternal age, a study conducted in India showed that mothers whose age is from 20 to 30 years and above 30 years were more likely to receive two doses of TT injection than mothers whose age is less than 20 years [7]. A similar study conducted in Kenya revealed that age group between 20 and 29 years was significantly associated with TT immunization status of mothers [8].

Studies revealed that mothers who had higher, secondary, and primary education were more likely to receive two or above doses of TT injections than mothers with no formal education [2, 7, 9], and concerning husband educational status, mothers whose husbands had primary and secondary and above education showed significant positive association with two or above doses of TT injections [10].

Regarding the occupational status of husband, mothers whose husbands are government employee were 75% more immunized than mothers whose husbands are other laborers and mothers who were government employee were 68.4% more immunized than mothers who were housewives [10].

Another cross-sectional study in Kenya revealed that mothers who make joint health decision with husband for their health issue were more likely to receive two doses of TT injection [11], and regarding wealth quintiles, mothers from richest family were more likely to have TT vaccination when compared to mothers from poorest family [6].

A study done in Lahore-Pakistan showed from the study participants 15% of mothers stated that TT vaccine was useless, 40% reasoned vaccination centers at a distant place, and 5% believed that it was harmful to the fetus [6]. A similar study conducted in Peshawar, Pakistan, showed that lack of awareness, distance, fear of side effect, and fear of sterility were reasons for not taking TT vaccination [10].

Cross-sectional studies conducted in Cameroon showed that knowledge and attitude of mothers were significantly associated with TT immunization status of mothers and mothers with higher knowledge had more potential than those with lower knowledge to get the complete TT immunization and mothers with a positive attitude for TT vaccination were more likely to obtain protective doses of TT injection compared to those with the negative attitude [12]. A similar study conducted in Pakistan showed that mothers' knowledge on TT vaccination had a significant and positive impact on TT immunization status and women having information about TT vaccination were more likely to receive TT vaccination as compared to their counterparts of having no information [13].

Studies carried out in Pakistan and Kenya revealed that mothers' antenatal care had significant and positive impact on TT immunization status of mothers and frequencies of health facility visit were significantly associated with TT immunization status [10, 11], and other cross-sectional studies in Kenya and Bangladesh showed that use of modern family planning method was significantly associated with TT immunization status [7, 11].

Cross-sectional study in Vientiane showed that parity, multipara mothers were more likely to use TT than primipara mothers [14], and another study in Bangladesh revealed that mothers who want more children in future were more likely to receive two or more doses of TT injection congruent parts [7].

A study done in Kenya showed that there was significant association between health information to the mothers and tetanus toxoid immunization coverage [8], and concerning the source for TT vaccination information, 33.6% participants heard from a health worker, 30.9% heard from school lessons, 13.8% heard from the mass media, 8.2% heard from the mass media and family members, and the remaining 6.2% heard from family members alone [15].

A study done in Ethiopia showed that nearly 74% of mothers had received at least two doses of TT injection [13]. According to 2011 DHS of Ethiopia, education and household wealth index have a positive effect on whether women receive TT injections. For example, 62 percent of mothers with more than secondary education received at least two injections during their last pregnancy compared with 29 percent of mothers with no education. Also, 52% of mothers in the highest wealth quintile received at least two doses of TT injection compared with 24 percent of mothers in the lowest wealth quintile [16], and according to 2016 EDHS, the proportion of mothers who protected against tetanus at last birth were 49% which was similar to that reported in the 2011 EDHS which was 48% [17].

Ethiopia has the highest neonatal tetanus morbidity and mortality rates in the world due to low TT immunization coverage coupled with the high amount of deliveries taking place at home in unsanitary conditions [18]. However, there has been no study conducted so far to assess factors associated with TT immunization status with the current global push for MNTE mothers with TT vaccination which is one of the proven strategies for eliminating MNT, towards reaching the MNT elimination goal. Thus, identifying factors influencing mothers from TT immunization is critical for countries like Ethiopia since institutional delivery is very low.

## 2. Method and Materials

### 2.1. The Study Area, Period, and Study Design

A community-based cross-sectional study was conducted from March 1 to 26, 2017, in Damboya Woreda, Kembata Tembaro Zone, South Ethiopia. Damboya is 16 km, 110 km, and 350 km away from zonal, regional, and federal head offices, respectively. The Woreda has 3 urban and 17 rural kebeles with the projected population of 105841 of whom 51862 (49%) were males. The total childbearing age women were 24,666, and estimated pregnancy was 3662 in the Woreda which constituted 3.5% of the population. According to Woreda Health Office Annual Report, the physical health services coverage was estimated to be 100%. There are four health centers and 19 health posts which are all government-owned and 2 medium clinics, 2 drug stores, and 1 diagnostic private-owned facility.

### 2.2. Population

#### 2.2.1. Source Population

All mothers who are in childbearing age were the source population in Damboya Woreda.

#### 2.2.2. Study Population

Sampled mothers who had given birth in the last 12 months in the selected kebeles during the data collection period were the study population.

### 2.3. Inclusion and Exclusion Criteria

Mothers who live in the Woreda for at least 6 months were included, and mothers who were critically ill or unable to respond were excluded from the study.

## 3. Sample Size Determination and Sampling Techniques

### 3.1. Sample Size Determination

The sample sizes were calculated for both single and double population proportion by manual for single population and using Epi InfoTM7 version 7.1 statistical functions for double population proportion.

- For single population proportion, the following parameters were considered:
   
*P* = proportion of mothers received at least two TT doses = 50.9% [17]
   
*D* = margin of error = 0.05 with 95% confidence interval
   
*Z* = 1.96 (level of significance)
   
*n* = (z*α*/2)2*p* (1 − *p*)/d2 = (1.96) 2.509 (1 – 0.509)/(0.05) 2 = 384
- For double population proportion calculated using Epi InfoTM7 version 7.1 Statcalc function.

Parameters used in double population proportion formula were as follows:

  P1 = % of outcome among the exposed group
  P2 = % of outcome among the unexposed group
  Confidence interval = 95%

The formula that yields the highest number was taken to calculate the final sample size. Considering the design effect of 2 and 10% nonresponse rate, the final sample size was 845 (Table 1).

### 3.2. Sampling Techniques

Two-stage stratified sampling technique was employed in the study. Total of 20 kebeles found in the Woreda were stratified into urban and rural kebeles. There were 3 urban and 17 rural kebeles. Then, 2 urban and 11 rural kebeles were selected by simple random sampling method. Registration of mothers who had given birth in the last 12 months was made from a family folder and delivery registration book at each health post level before the actual data collection in all the selected kebeles to create sampling frame. Then each household was given consecutive corresponding house number according to the sampling frame. If there is more than one mother in the household with a history of birth in the last 12 months, a mother was selected by lottery method. The number of study participants from each selected kebele was determined based on the number of childbearing age mothers in each kebele. Finally, a simple random sampling technique was employed to select the study participants.

## 4. Variables

### 4.1. Dependent Variable

TT immunization status.

### 4.2. Independent Variables

Sociodemographic characteristics: age, marital status, religion, ethnicity, educational status of the mother, educational status of husband, wealth index of household, mother occupation, husband occupation, use of mass media, place of residence, and making a joint decision with husband for a health issue. Reproductive factors: parity and pregnancy plan. Behavioral factors: knowledge, attitude, ANC visit, and use of modern family planning method. Health service-related factors: accessibility of the service, health extension worker home visit, and getting information for TT vaccination from health professionals.

### 4.3. Operational Definitions

Immunization status: when mothers had received <2 TT dose (not protected at birth) or had received ≥2 TT doses (protected at birth).

Protected at birth (PAB): if the mother had received documented or not, two or more doses during current pregnancy or at least two TT doses prior to the current pregnancy of which the last dose was <3 years before the birth; or three doses within the 5 years before the current pregnancy; or four doses with the last dose <10 years before the pregnancy or receiving five doses or more before the current pregnancy.

### 4.4. Instruments

Data were collected by using structured questionnaire adapted from WHO, EDHS, and different kinds of literature and adapted to the local context. The structured questionnaire was prepared in English, translated to Amharic and Kembatissa, and back-translated to the English language by independent translators.

### 4.5. Data Collection Process

Participants were interviewed in their household using translated structured questionnaire. Data collection was carried out by 13 trained diploma nurses who speak Amharic and Kembatissa fluently with 13 local guides to simplify the data collection process. Information on socioeconomic and demographic characteristics, reproductive characteristics, health service-related factors, and behavioral factors was collected with immunization history of mothers which was collected from vaccination cards, mother's verbal report, or both.

### 4.6. Data Processing and Analysis

Data were checked, cleaned, and entered into Epi data software version 3.1 and then exported to SPSS version 22 for analysis. Descriptive statistics were used to summarize the data. Bivariate logistic regression analysis was done to identify candidates for multivariable logistic regression analysis. All explanatory variables with a *p* value of less than 0.2 in bivariate logistic regression analysis were included in the initial logistic model of multivariable logistic regression analysis to identify independent predictors. Then crude and adjusted odds ratio together with their corresponding 95% confidence intervals were computed. A *p* value < 0.05 was considered as statistically significant in this study.

### 4.7. Data Quality Assurance

To assure the quality of data, the questionnaire was translated to Amharic and Kembatissa languages and back-translated into English to see the consistency. Both the interviewers and supervisors were trained for two days on the objectives, data collection, and interviewing approaches. A pretest was conducted in 5% of the samples of both urban and rural kebeles. The reliability test was conducted and the value of Cronbach's alpha > 0.7 items is considered as reliable for study questionnaire. Completeness, consistency, and applicability of the instruments were ratified accordingly. At the time of data collection, filled questionnaires were checked for completeness and consistency of information by the PI and supervisor on daily basis.

## 5. Results

### 5.1. Socioeconomic and Demographic Characteristics

Of the total 845 mothers planned to be included in the study, 837 mothers who had given birth in the last 12 months before the survey were actually participated in the study by giving 99% response rate.

Based on residence, 710 (84.8%) rural and 510 (60.1%) of the mothers age falls in the range of 21–30 years with the mean age of 28 (±5.362) years. Seven hundred ninety-one (94.5%) were Kembata by ethnicity, 699 (83.5%) were protestant religion followers, and 822 (98.2%) were married. Regarding educational status, 384 (45.9%) mothers and 517 (61.8%) mother's husband have no formal education, respectively, and 788 (94%) mothers were housewife by occupation; likewise, 79.9% husbands were farmers. Regarding family size, 524 (62.5%) mothers had less than or equal to five family members, and concerning mass media, 35.5% and 4.4% mothers were an owner of radio and television, respectively (Table 2).

### 5.2. Reason for Not Starting or Completing TT Vaccination

**Figure 1:**
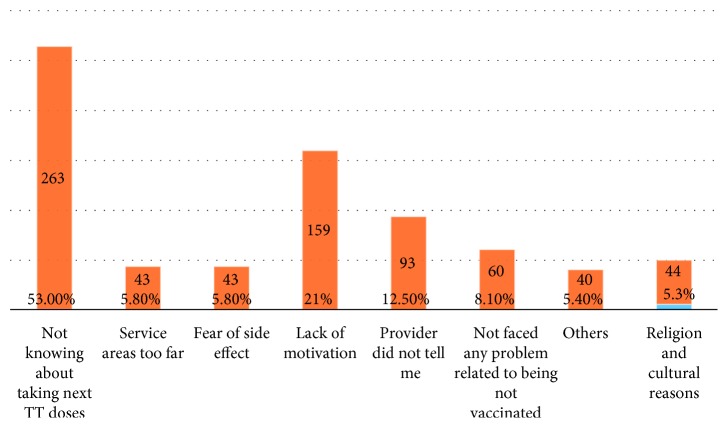
Reasons given by mothers for not completing TT vaccination in Damboya Woreda, South Ethiopia (*n*=745).

**Figure 2:**
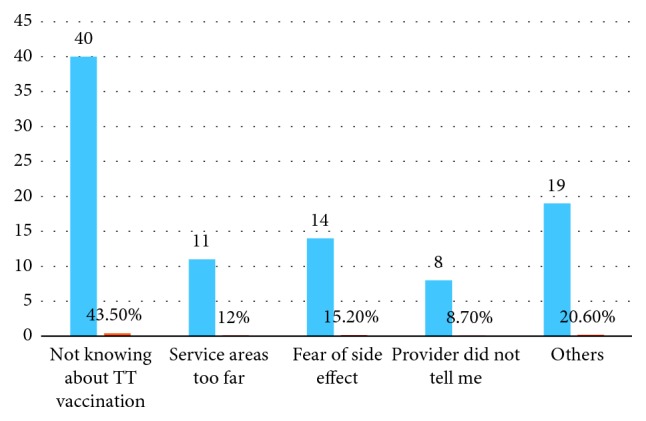
Reasons explained by mothers for not started TT vaccination in Damboya Woreda South Ethiopia (*n*=92).

As mothers were asked for the reasons why they either do not complete or started tetanus toxoid vaccination, the response was not knowing about taking next dose of TT vaccine (263, 53%), lack of motivation to go for next doses (159, 21%), and no problem faced related to being not immunized (60, 8.1%) were the main reasons for not completing and not knowing about TT vaccination (40, 43.5%) was main reasons for not starting TT vaccination (Figures 1 and 2).

### 5.3. Reproductive Characteristics of Mothers

Five hundred four (60.2%) women gave birth to 2–4 children in their life. The majority (86.9%) of mothers had planned to have a child for last pregnancy and 87.7% mothers have future fertility plan (Table 3).

### 5.4. Behavioral Factors on Tetanus Toxoid Immunization Status

Concerning behavioral factors, 784 (93%) mothers attended at least one antenatal care visit, 566 (67.6%) mothers used modern family planning method, and 320 (38.2%) had poor knowledge with a mean score of 7.7 (±2.3) (Table 4).

### 5.5. Health Service-Related Factors on Tetanus Toxoid Immunization Status

Seven hundred seven (84.5%) mothers had obtained TT vaccine information from health professionals. For accessibility of TT vaccination services, 814 (97.3%) mothers said TT vaccination was provided by facility nearby and 767 (91.6%) of them walk less than 1 hour on foot to reach the nearest health facility. 448 (53.5%) mothers had vaccination card, while the rest 389 (46.5%) did not have during survey time. Majority of 614 (73.4%) mothers were visited by health extension package workers in their home in last pregnancy time (Table 5) and 463 (55.3%) health extension package workers and 165 (23%) health workers were their source of information (Table 6).

### 5.6. Immunization Status of Mothers

**Figure 3:**
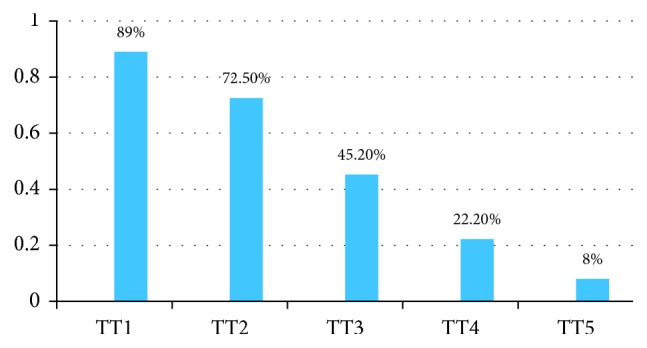
Level of tetanus toxoid vaccination coverage of mothers in Damboya Woreda, South Ethiopia, March 2017 (*n*=745).

Based on mother's vaccination card and oral history, 607 (72.5%) mothers were protected against tetanus at last birth. For 448 (53.5%) mothers, the source for their tetanus toxoid immunization status was card plus oral history, while for the rest 389 (46.5%) the source was oral history only (Figure 3).

### 5.7. Factors Associated with TT Immunization Status of Mothers

Variables in the bivariate analysis with a *p* value < 0.2 were included in the multivariable analysis to determine independent predictors of TT immunization status.

Age of mother, mothers' educational status, husbands' educational status, making joint decision with husband for their health issue, health extension worker home visiting, use of modern family planning method, number of antenatal care visit, and time to reach the nearest health facility were independent predictors of TT immunization status of mothers with *p* value < 0.05 (Table 5).

## 6. Discussion

In this study, immunization status was assessed using the availability of vaccination card and maternal recall (oral history). Based on both immunization card and oral history, 607 (72.5%) mothers were protected against tetanus at their last birth. This finding is similar to a study done in Ethiopia and Ghana [4, 9] but higher than the proportion reported in 2016 EDHS report which was 49% and 50.9% for national and SNNPR, respectively [11]. This difference might be due to hard to reach areas included in EDHS report and the additional health center construction in Damboya Woreda that gives an extra opportunity for TT vaccination.

This study showed that the major reasons for incomplete TT vaccination were as follows: mother not knowing about taking the next dose of TT vaccination, lack of motivation to receive the next dose, and provider did not tell me during facility visit while not knowing about TT vaccination, services area too far, and fear of side effect, which is in line with the studies conducted in Kenya and Pakistan [7, 8]. This implies that utilization of TT vaccination service is dependent on mother's understanding of its importance and will be improved by conducting regular and focused education and communication activities on the need for vaccination, as well as interpersonal communication and negotiation on the need for subsequent doses of vaccine.

Also, this study revealed that mothers whose age is between 21 and 30 and >30 years were 3.36 and 9.69 times more likely to receive two doses of TT injection than mothers whose age is ≤ 20 years, respectively. This finding is similar to study findings in Kenya, Vientiane, and India [6, 8, 14]. This is probably because of this age group actively participated in different health-related issues and their age maturity concerning marriage in taking responsibility for child health care. In addition, this age group may have past exposure which gave them practical knowledge about healthcare services to themselves and their children.

Concerning mothers' educational status, mothers who attended elementary school were 3.72 times more likely to receive two doses of TT injection than mothers who have no formal education. This is similar to the study done in Ethiopia and Bangladesh [6, 10]. This may be due to easy access to information and knowledge of a mother on immunization services and because of the fact that education is likely to enhance confidence.

Mothers whose husband had elementary and secondary and above education were 1.84 and 4.13 times more likely to receive two doses of TT injection, respectively, compared to mothers whose husband had no formal education. This finding is in line with the study done in Asia, Bangladesh, and Pakistan [2, 6, 7]. This may be due to the fact that educated husband has more exposure to different information sources concerning health issues and better level of understanding than the noneducated husband. Also, it is a general fact that education increases overall awareness including health and healthcare utilization of an individual.

The finding of this study revealed that mothers who attended 2-3 antenatal care visits were 4.68 times more likely to receive two doses of TT injection than mothers who attended less than two antenatal care visits. This finding is in line with the study conducted in Kenya, Vientiane, and Pakistan [7, 8, 14]. This might be because repeated health facility visit for ANC follow-up gives an opportunity for TT vaccine injection and provides an insight about NNT.

Regarding distance from home to the nearest health facility, mothers who walk less than 1 hour to reach the nearest health facility were 2.93 times more likely to receive two doses of TT injection than mothers who walk greater than or equal to 1 hour to reach the nearest health facility. This finding is similar to the study done in Ethiopia [9]. And it indicates that as the time to reach health facility increases, the accepted rate of TT vaccine decreases and increasing accessibility of TT vaccination providing facility is good.

Mothers who use modern family planning method were 5.19 times more likely to receive two doses of TT injection as compared with those who do not use modern family planning. This is similar to the study conducted in Bangladesh [6]. It might be because health information is given in family planning service due to service integration.

Health extension worker home visit had positive impact on TT immunization status of a mother and a mother who was visited in her home during last pregnancy time by health extension worker was 7 times more likely to receive two doses of TT injection than a mother who did not visit. This is similar to the study done in Pakistan [7]. This might be because of the health education provided by health extension worker regarding the advantages of having TT vaccine during pregnancy and encouraging the mother to follow ANC as well.

Another predictor was making a joint decision with the husband about their health issue. In this study, a mother who made a joint decision about her health issue with her husband was 4 times more likely to receive two doses of TT injection than a mother who did not make a joint decision with her husband or made health decision alone. This finding is in line with the study done in Kenya [8]. This might be because a mother who made joint discussion with husband gets more confident and social support for the TT utilization and will have protective doses of TT vaccine.

### 6.1. Strength

Selection bias was minimized since it was community-based study with probability sampling technique. Gender match interview was conducted, and all the interviewers were familiar with the local language.

### 6.2. Limitation

Since immunization status was collected including self-report of mothers, this made it susceptible for recall bias. Institutional card or registration book review was not conducted to reduce recall bias.

## 7. Conclusion and Recommendations

In general, this study concluded that near to three-fourth of the mothers were protected against tetanus in last birth which was very low as to WHO recommendation for pregnant mothers (100%). Also, the study found that mother's age, mother's educational status, husband educational status, use of modern family planning method, number of antenatal care visit, time to reach the nearest health facility, health extension home visit, and making joint decision with husband for their health issue were independent predictors for TT immunization status of mothers.

### 7.1. For Policy and Practice

Even though most mothers had access for TT immunization service, they were not immunized with protective doses of TT vaccine. So, at a community level joint decision making of mothers and husbands has to be strengthen to enhance health-seeking behavior and to increase the proportion of mothers protected at birth. It is also very important to work on health extension workers' commitment level improving programmed home visit services to pregnant mothers and strengthen mother's facility visit for ANC follow-up. Convenient outreach sites have to be established for those who are living distant from health facility to provide intensive public health education to fill the knowledge gap among the mothers.

### 7.2. For Future Research

This study provides an opportunity to assess TT immunization status and its predictors among mothers. In this regard, other studies should be conducted aiming at determining the proportion of tetanus toxoid immunization status of mothers by antibody testing to know the absolute proportion of mothers protected tetanus at birth.

## Figures and Tables

**Table 1:** List of exposure variables to calculate sample size for associated factors.

Exposure variable	% of outcome among the unexposed group	% of outcome among the exposed group	Odds ratio (OR)	Confidence interval (%)	Power	Sample size (*n*)	References
Maternal education (secondary and above)	41	83		95	80	50	[11]
ANC visit (2 and above)	45.3	81.9	2.14	95	80	64	[14]

**Table 2:** Socioeconomic and demographic characteristics of mothers, in Damboya Woreda, South Ethiopia, March 2017 (*n*=837).

Variables	Number of mothers	Percentage
*Residence*		
Rural	709	84.8
Urban	128	15.2

*Age*		
≤20 years	**113**	**13.5**
21–30 years	**510**	**60.9**
>30 years	**214**	**25.6**

*Ethnicity*		
Kembata	791	94.5
Hadiya	26	3.1
Others	20	2.4

*Religion*		
Protestant	699	83.5
Muslim	76	9.1
Orthodox	46	5.5
Catholic	16	1.9

*Marital status*		
Married	822	98.2
Others	15	1.8

*Mother educational status*		
No formal education	384	45.9
Elementary school	377	45
Secondary and above	76	9.1

*Husband educational status*		
No formal education	517	61.8
Elementary school	265	31.7
Secondary and above	55	6.5

*Mother occupational status*		
Housewife	788	94.2
Merchant	22	2.6
Others	27	3.2

*Husband occupational status*		
Farmer	669	79.9
Daily laborer	91	10.9
Government employee	37	4.4
Merchant	31	3.7
Others	9	1.1

*Wealth index of household*		
Lowest	168	20
Second	167	20
Middle	167	20
Fourth	168	20
Highest	167	20

*Household size*		
Less than or equal to 5	524	62.5
Greater than 5	313	37.5

*Having radio*		
Yes	295	35.5
No	542	64.5

*Having television*		
Yes	37	4.4
No	800	95.6

*Making a joint decision with husband about the health issue*		
Yes	680	81.25
No	157	18.75

**Table 3:** Frequency of selected reproductive factors of mothers in Damboya Woreda, March 2017 (*N*=837).

Variables	Category	≥2 TT injections	<2 TT injections	COR (95% CI)
Parity	1	89 (51.1%)	85 (48.9%)	1
	2–4	389 (77.2%)	115 (22.8%)	3.23 (2.25–4.64)
	≥5	129 (81.1%)	30 (18.9%)	4.11 (2.5–6.75)

Future fertility plan	Yes	531 (72.3%)	203 (27.7%)	1.05 (0.67–1.64)
	No	75 (74.3%)	26 (25.7%)	1

Plan for last childbirth	Yes	568 (78.1%)	159 (21.9%)	6.5 (4.24–9.98)
	No	39 (35.5%)	71 (64.5%)	1

**Table 4:** Frequency of knowledge response of mothers on tetanus and TT vaccine, in Damboya Woreda, South Ethiopia, March 2017 (*N*=837).

Items	Categories	Number of mothers	Percentage
Have you ever heard of about TT vaccination?	Yes	749	89.5
	No	88	10.5
Lockjaw is the symptom of tetanus	Yes	603	72
	No	224	28
Neck stiffness is a symptom of tetanus	Yes	620	74.1
	No	117	25.9
Tetanus toxoid vaccine prevents tetanus	Yes	640	76.5
	No	197	23.5
Tetanus toxoid vaccines serve as family planning	Yes	85	10.2
	No	752	89.8
Tetanus toxoid vaccine gives protection for only mothers	Yes	109	13
	No	628	87
Only one tetanus toxoid injection not gives protection	Yes	451	53.9
	No	386	46.1
Five times TT injection give lifelong protection	Yes	567	67.7
	No	270	37.3
Tetanus toxoid injection gives protection for both mothers and baby from tetanus during birth	Yes	643	76.8
	No	194	23.2

**Table 5:** Independent predictors of the tetanus toxoid immunization status of mothers in Damboya Woreda, South Ethiopia, March 2017 (*n*=837).

Variables	≥2 TT injections, *n* (%)	<2 TT injections, *n* number (%)	AOR	95% CI
*Age*				
≤20 years	33 (29.2%)	80 (70.8%)	1	
21–30 years	392 (76.9%)	118 (23.1%)	4.61	2.26–9.41^*∗*^
≥31 year	182 (85%)	32 (15%)	9.69	4.11–22.84^*∗*^

*Mother's educational status*				
No formal education	207 (53.9%)	177 (46.1%)	1	
Elementary school	334 (88.6%)	43 (11.4%)	3.42	2–5.86^*∗*^
Secondary and above	66 (86.8%)	10 (13.2%)	1.95	0.75–5.1^*∗*^

*Husband's educational status*				
No formal education	333 (64.4%)	184 (35.6%)	1	
Elementary school	227 (85.7%)	38 (14.3%)	1.84	1.03–3.28^*∗*^
Secondary and above	47 (85.5%)	8 (14.5%)	4.13	1.07–5.07^*∗*^

*Using modern family planning*				
Yes	467 (82.5%)	99 (17.5%)	5.16	3.09–8.59^*∗*^
No	140 (51.7%)	131 (48.3%)	1	

*Making a joint decision with the husband for health issues*				
Yes	526 (81.9%)	116 (18.1%)	4.25	2.45–7.32^*∗*^
No	81 (41.5%)	114 (58.5%)	1	

*Number of antenatal care visit*				
1	50 (38.8%)	79 (61.2%)	1	
2–3	443 (82%)	97 (18%)	4.1	2.28–7.37^*∗*^
≥4	103 (89.6%)	12 (10.4%)	10.09	3.63–28.1^*∗*^

*Planned for last child pregnancy*				
Yes	568 (78.1%)	159 (21.9%)	0.07	0.94–3.85
No	39 (35.5%)	71 (64.5%)	1	

*Health extension package worker home visit*				
Yes	535 (87.1%)	79 (12.9%)	6.51	3.78–11.19^*∗*^
No	72 (32.3%)	151 (67.7%)	1	

*Time to reach the nearest health facility from home on foot*				
<1 hour	571 (74.4%)	196 (25.6%)	3.61	1.66–7.84^*∗*^
≥1 hour	36 (51.4%)	34 (48.6%)	1	

**Table 6:** Frequency of health service-related factor on tetanus toxoid immunization status mothers, Damboya Woreda, South Ethiopia, March 2017 (*n*=837).

Variables (*N*=837)	Category	Number	Percentage
Traveling time (in hour) from home to the nearest health facility	Less than 1 hour	767	91.6
	≥1 hour	70	8.4

Accessibility of TT vaccination service	Yes	814	97.3
	No	23	2.7

Getting TT vaccination information from health professionals	Yes	707	84.5
	No	130	15.5

Visited by health extension package worker at home	Yes	614	73.4
	No	223	26.6

## Data Availability

The data used to support the findings of this study are available from the corresponding author upon request.

## Abbreviations

AOD: Adjusted odds ratio
CI: Confidence interval
COR: Crude odds ratio
EDHS: Ethiopian Demographic Health Survey
IRB: Institutional review board
MNET: Maternal and Neonatal Tetanus Elimination
NT: Neonatal tetanus
SPSS: Statistical Package for Social Sciences
TT: Tetanus toxoid
WHO: World Health Organization.

## Supplementary Materials

Supplementary Materials Table S1: bivariate analysis of socioeconomic and demographic characteristics of mothers on tetanus toxoid immunization status in Damboya Woreda, March 2017 (*n* = 837). Table S2: bivariate analysis of behavioral factors on the tetanus toxoid immunization status of mothers in Damboya Woreda, South Ethiopia, March 2017 (*n* = 837). Table S3: bivariate analysis of service-related factors on the tetanus toxoid immunization status of mothers, Damboya Woreda, South Ethiopia, March 2017 (*n* = 837).

## References

[1] Anokye M., Mensah J. A., Frimpong F., Aboagye E., Acheampong N. (2014). Immunization coverage of pregnant women with tetanus toxoid vaccine in dormaa east district-brong adaro region, Ghana. *Mathematical Theory and Modeling*.

[2] Rana Anglican E., Raza M. A. (2013). Maternal health care: the case of tetanus toxoid vaccination. *Asian Development Policy Review*.

[3] UNICEF (2010). *Achieving and Sustaining Maternal and Neonatal Tetanus Elimination Strategic Plan 2012-2015*.

[4] WHO (2017). *Maternal and Neonatal Tetanus Elimination the Initiative and Challenges Why Maternal and Neonatal Tetanus Elimination*.

[5] Diamenu S. K., Bosnu G., Abotsi F. (2015). Introducing protection at birth method of monitoring tetanus-diphtheria vaccination coverage of mothers in Ghana. *International Journal of Vaccines and Immunization*.

[6] Hashmi F. K., Islam M., Khan T. A., Tipu M. K. (2011). Vaccination coverage of mothers during pregnancy with tetanus toxoid and infants after birth. *Pakistan Journal of Pharmacy*.

[7] Singh A., Pallikadavath S., Ogollah R., Stones W. (2012). Maternal tetanus toxoid vaccination and neonatal mortality in Rural North India. *PLoS One*.

[8] Naeem M., Khan M. Z., Abbas S. H. (2010). Coverage, and factors associated with TT vaccination among married women of reproductive age, Peshawar Pakistan. *Journal of Ayub Medical College*.

[9] Haile Z. T., Azulay Chertok I. R., Teweldeberhan A. K. (2013). Determinants of utilization of sufficient TT immunization during pregnancy: evidence from the Kenya demographic and health survey, 2008-2009. *Journal of Community Health*.

[10] JSI (2015). *An Extended Programme on Immunization Coverage in Selected Ethiopia Zones a Baseline Survey for L10 kms Routine Immunization Improvement Initiative*.

[11] Agency C. S. (2011). *Ethiopia Demographic and Health Survey Addis Ababa*.

[12] Agency C. S. (2016). *Ethiopia Demographic and Health Survey Addis Ababa*.

[13] EMO Health (2010). *National Expanding Programme of Immunization*.

[14] Masuno K., Xaysomphoo D., Phengsavanh A., Douangmala S., Kuroiwa C. (2009). Scaling up interventions to eliminate neonatal tetanus: factors associated with the coverage of tetanus toxoid and clean deliveries among women in Vientiane, Lao PDR. *Vaccine*.

[15] Alex-Hart B., Okoh B. (2015). Awareness and status of tetanus toxoid vaccination among female undergraduate students in a Nigerian University. *International Journal of Tropical Disease & Health*.

[16] Ngachangong V. M., Melanie M. G., Tufon E. N. (2014). Factors related to the escapement of reproductive age women from tetanus toxoid vaccination at the sub-divisional medicalized health center, Nkwen, Bamenda Cameron. *Vedic Research International Cell Signaling*.

[17] Debbie A., Amdu A., Alamneh A. (2016). A clinical profile of tetanus patients attended at felegehiwot referral hospital, northwest ethiopia: a retrospective cross-sectional study. *SpringerPlus*.

[18] Maima W., Ephantus M., Kabiru E. A. (2014). Utilization of antenatal TT immunization services among women in Bahati division, Naku country Kenya. *International of innovative research and studies*.

