# Supplementary material for: Tetanus Toxoid Immunization Status and Associated Factors among Mothers in Damboya Woreda, Kembata Tembaro Zone, SNNP, Ethiopia
Source: J Nutr Metab. 2018 Nov 22;2018:2839579. doi: 10.1155/2018/2839579 (PMC6282149; doi:10.1155/2018/2839579)
Supplement: Supplementary Materials — Table S1: bivariate analysis of socioeconomic and demographic characteristics of mothers on tetanus toxoid immunization status in Damboya Woreda, March 2017 (n = 837). Table S2: bivariate analysis of behavioral factors on the tetanus toxoid immunization status of mothers in Damboya Woreda, South Ethiopia, March 2017 (n = 837). Table S3: bivariate analysis of service-related factors on the tetanus toxoid immunization status of mothers, Damboya Woreda, South Ethiopia, March 2017 (n = 837). [file 2839579.f1.doc]

Table S1: Bivariate analysis of socioeconomic and demographic characteristics of mothers on tetanus toxoid immunization status in Damboya Woreda, March 2017 ($n=837$).

| Variables | ≥2 TT injection number (%) | <2 TT injection number (%) | COR | 95% CI |
| --- | --- | --- | --- | --- |
| *Residence* | | | | |
| Rural | 518 (73.1%) | 191 (26.9%) | 1 |  |
| Urban | 89 (69.5%) | 39 (30.5%) | 1.188 | 0 .79–1.79 |
| *Age* | | | | |
| ≤20 years | 33 (29.2%) | 80 (70.8%) | 1 |  |
| 21–30 years | 392 (76.9%) | 118 (23.1%) | 8.05 | 5.1–12.7 |
| ≥31 year | 182 (85%) | 32 (15%) | 13.78 | 7.93–23.97 |
| *Marital status* | | | | |
| Married | 595 (72.4%) | 227 (27.6%) | 0.66 | 0.18–2.33 |
| Others^1^ | 10 (66.6%) | 5 (33.3%) | 1 |  |
| *Mother’s educational status* | | | | |
| No formal education | 207 (53.9%) | 177 (46.1%) | 1 |  |
| Elementary school | 334 (88.6%) | 43 (11.4%) | 6.64 | 4.56–9.67 |
| Secondary and above | 66 (86.8%) | 10 (13.2%) | 5.64 | 2.82–11.3 |
| *Husband’s educational status* | | | | |
| No formal education | 333 (64.4%) | 184 (35.6%) | 1 |  |
| Elementary school | 227 (85.7%) | 38 (14.3%) | 3.3 | 2.24–4.87 |
| Secondary and above | 47 (85.5%) | 8 (14.5%) | 3.25 | 1.5–7.02 |
| *Mother’s occupation* | | | | |
| Housewife | 566 (71.8%) | 222 (28.2%) | 1 |  |
| Others^2^ | 41 (83.7%) | 8 (16.3%) | 2 | 0.93–4.36 |
| *Husband’s occupation* | | | | |
| Farmer | 489 (72.8%) | 183 (27.2%) | 1 |  |
| Others^3^ | 118 (71.5%) | 47 (28.5%) | 0.94 | 0.644–1.37 |
| *Having radio* | | | | |
| Yes | 213 (72.2%) | 82 (27.8%) | 0.88 | 0.71–1.34 |
| No | 394 (72.7%) | 148 (27.3%) | 1 |  |
| *Making a joint decision with husband for health issues* | | | | |
| Yes | 526 (81.9%) | 116 (18.1%) | 6.38 | 4.5–9 |
| No | 81 (41.5%) | 114 (58.5%) | 1 |  |
| *Household size* | | | | |
| ≤5 | 378 (71.4%) | 146 (28.6%) | 1 |  |
| >5 | 229 (73.2%) | 84 (26.8%) | 0.95 | 0.69–1.3 |

*Note*. Significant at 95% confidence interval (CI), ^1^others = windowed, divorced, and never married, ^2^others = daily laborer and government employee, ^3^others = daily laborer, government employee, merchant, and not employed. COR = crude odds ratio.

Table S2: Bivariate analysis of behavioral factors on the tetanus toxoid immunization status of mothers in Damboya Woreda, South Ethiopia, March 2017 ($N=837$).

| Variables | Category | ≥2 TT injections, *n* (%) | <2 TT injections, *n* (%) | COR (95% CI) |
| --- | --- | --- | --- | --- |
| Knowledge level | Poor | 192 (67.8%) | 91 (32.2%) | 1 |
|  | Good | 415 (74.9%) | 139 (25.1%) | 1.42 (1.04–1.94) |
| Using modern family planning | Yes | 467 (82.5%) | 99 (17.5%) | 4.41 (3.1–6.1) |
|  | No | 140 (51.7%) | 131 (48.3%) | 1 |
| Number of ANC visit | 1 | 50 (38.8%) | 79 (61.2%) | 1 |
|  | 2–3 | 443 (82%) | 97 (18%) | 7.22 (4.76–10.95) |
|  | ≥4 | 103 (89.6%) | 12 (10.4%) | 13.56 (6.77–27.2) |

Table S3: Bivariate analysis of service-related factors on the tetanus toxoid immunization status of mothers, Damboya Woreda, South Ethiopia, March 2017 ($N=837$).

| Variables | Category | ≥2 TT injections, *n* (%) | <2 TT injections, *n* (%) | COR (95% CI) |
| --- | --- | --- | --- | --- |
| Time to reach nearest health facility from home on foot | <1 hr | 571 (74.4%) | 196 (25.6%) | 2.59 (1.53–4.39) |
|  | ≥1 hr | 36 (51.4%) | 4 (48.6%) | 1 |
| Getting TT vaccination information from health professionals | Yes | 534 (75.5%) | 173 (24.5%) | 2.4 (1.64–3.55) |
|  | No | 73 (56.2) | 57 (43.8%) | 1 |
| Visited by health extension package worker at home | Yes | 535 (87.1%) | 79 (12.9%) | 14.2 (9.85–20.51) |
|  | No | 140 (51.7%) | 131 (48.3%) | 1 |
